# Recent update on biological activities and pharmacological actions of liraglutide

**DOI:** 10.17179/excli2017-323

**Published:** 2017-05-17

**Authors:** Juhi Tiwari, Gaurav Gupta, Rajiv Dahiya, Kavita Pabreja, Rakesh Kumar Sharma, Anurag Mishra, Kamal Dua

**Affiliations:** 1 School of Pharmacy, Jaipur National University, Jagatpura 302017, Jaipur, India; 2 School of Medicine and Public Health, University of Newcastle, Newcastle, NSW 2308, Australia; 3 Laboratory of Peptide Research and Development, School of Pharmacy, Faculty of Medical Sciences, The University of the West Indies, St. Augustine, Trinidad & Tobago, West Indies; 4 School of Pharmacy, Suresh Gyan Vihar University, Jagatpura 302017, Jaipur, India; 5 Discipline of Pharmacy, Graduate School of Health, University of Technology Sydney, Sydney, NSW 2007, Australia; 6 School of Biomedical Sciences and Pharmacy, University of Newcastle, Newcastle, NSW 2308, Australia; 7 School of Pharmaceutical Sciences, Shoolini University, Solan, Himachal Pradesh, 173229, India

## ⁯

Dear Editor, 

Liraglutide (LG), an analog of human glucagon-like peptide 1 (GLP-1), has been permitted for type 2 diabetes therapy. LG triggers the GLP-1 receptor, leading to release of insulin in the presence of high glucose concentrations, it declines secretion of glucagon in a glucose-dependent manner and directly applying to the β cells of pancreas to help its proliferation and differentiation (Dharmalingam et al., 2011[11]; Drucker et al., 2010[12]). The mechanism of lowering blood glucose also includes a delay in gastric emptying. LG is approved by the European Medicines Agency (EMA) on July 3, 2009, and by the U.S. Food and Drug Administration (FDA) on January 25, 2010, for the treatment of type 2 diabetes mellitus (T2DM) (Ye et al., 2017[48]). A number of studies suggest that LG, GLP-1 has additional benefits (Zhang et al., 2017[49]). Here we have reviewed various pharmacological actions of LG (Table 1(Tab. 1)). (References in Table 1: Li et al., 2017[32]; Qi et al., 2017[43]; Feng et al., 2017[13]; Garg et al., 2017[14]; King et al., 2017[26]; Hu et al., 2017[16]; Badawi et al., 2017[4]; le Roux et al., 2017[30]; Mezquita-Raya et al., 2017[39]; Martinez et al., 2017[37]; Anholm et al., 2017[2]; Kumarathurai et al., 2017[27]; Hunt et al., 2017[18]; Iacobellis et al., 2017[20]; von Scholten et al., 2017[45]; Manigault and Thurston, 2016[35]; Palleria et al., 2017[40]; Ishii et al., 2017[21]; Bisgaard et al., 2016[5]; Abdelsameea et al., 2017[1]; Jennings et al., 2016[22]; Chen et al., 2016[9]; Dejgaard et al., 2017[10]; Bouchi et al., 2017[6]; Li et al., 2016[33]; Hvistendahl et al., 2016[19]; Bouchi et al., 2017[6]; Arturi et al., 2016[3]; Kumarathurai et al., 2017[28]; Ke et al., 2016[25]; Zhou et al., 2016[50]; Zobel et al., 2017[51]; Calvo Gomez et al., 2016[7]; Petit et al., 2017[41]; Kaur et al., 2016[24]; Mansur et al., 2017[36]; Wang et al., 2016[46]; Hu et al., 2016[17]; Lee et al., 2016[31]; Pra et al., 2016[42]; Langlois et al., 2016[29]; Mathieu et al., 2016[38]; Wang et al., 2016[47]; Saponaro et al., 2016[44]; Li et al., 2016[34]; Htike et al., 2016[15]; Kato et al., 2016[23]; Chai et al., 2016[8].)

| Key Findings | References |
|---|---|
| By targeting insulin receptor substrate 1 on the diabetic rat pancreas and insulin 1 cells, LG exhibits anti-apoptotic effect by decreased miR-139-5p expression. | Li et al., 2017 |
| By declining the phosphorylation of tau, LG alleviates Alzheimer disease-like cognitive impairment. | Qi et al., 2017 |
| In T2DM patients with non-alcoholic fatty liver disease, compare to metformin and gliclazide, LG showed a better recovery in hepatic function, decreases in integration host factor content and level of HbA1c, and reduction in weight. | Feng et al., 2017 |
| LG in a one dose can acutely inhibit the ketogenesis. | Garg et al., 2017 |
| Therapy with a combination of Insulin Degludec (IDeg) and LG (IDegLira) allows more patients with type 2 diabetes to sustain the level of blood glucose within target ranges all over the day compared to IDeg or LG alone. | King et al., 2017 |
| LG can protect cardiomyocytes against reperfusion injury through modulation of intracellular calcium homeostasis. | Hu et al., 2017 |
| LG and sitagliptin showed an auspicious approach to moderate the progression of Parkinson's disease by their anti-inflammatory, anti-apoptotic neurotrophic and neurogenic mechanistic activities. | Badawi et al., 2017 |
| Treatment of LG 3.0 mg may deliver benefit for health in terms of reduced risk of diabetes in persons with obesity and prediabetes. | le Roux et al., 2017 |
| LG 1.8 mg is likely to be cost-effective compared with lixisenatide 20 µg in T2D patients who have not attained glycemic control targets on metformin monotherapy. | Mezquita-Raya et al., 2017 |
| LG therapy may help patients in primary care by postponing the need for further treatment strengthening. | Martinez et al., 2017 |
| LG accompanying with metformin induces a significantly clinical enhancement in β-cell function in obese and patients with cardiovascular diseases with newly diagnosed well-controlled T2DM and coronary artery diseases. | Anholm et al., 2017 |
| LG showed daytime variation in the effect on blood pressure without upsetting the blood pressure inconsistency or night-time blood pressure dropping. | Kumarathurai et al., 2017a |
| Treatment of T2DM patients with LG 1.8 mg is expected to be measured highly cost-effective compared with lixisenatide 20 µg in the UK setting. | Hunt et al., 2017 |
| LG causes a considerable and quick EAT reduction. Liraglutide cardiometabolic effects may be EAT-mediated. | Iacobellis et al., 2017 |
| LG treatment showed anti-inflammatory, reflected in decreases in TNF-α and MR-proADM, whereas a decrease in MR-proANP may signify a clinically relevant regarding cardiac failure. | von Scholten et al., 2017 |
| LG 3.0 mg treatment showed safe and effective addition to the pharmacologic effect for chronic weight management in the overall population. | Manigault and Thurston, 2016 |
| Through an effect on mTOR pathway, LG protected diabetes-dependent hippocampal neurodegeneration. | Palleria et al., 2017 |
| In obese patients with T2D, LG expressively enhanced glycemic control and decreased the body weight without failing quality of life. | Ishii et al., 2017 |
| LG has valuable effects in a mouse model of moderate uremia by decreasing atherosclerosis and inflammation of kidney. | Bisgaard et al., 2016 |
| LG defends in contrast to IR injury of the hepatic tissue through anti-inflammatory and antioxidant activities along with inhibition of apoptosis. | Abdelsameea et al., 2017 |
| IDegLira provided superior glycemic control versus unchanged GLP-1RA and represents an efficacious intensification approach in patients inadequately controlled on GLP-1Ras. | Jennings et al., 2016 |

*EXCLI Journal 2017;16:742-747 – ISSN 1611-2156*
*Received: April 01, 2017, accepted: May 03, 2017, published: May 17, 2017*

| Key Findings | References |
|---|---|
| LG treatment progresses myocardial recovery and infarct size after ST-segment-elevation myocardial infarction, possibly by reducing reperfusion injury, making it a promising treatment for evaluation in larger trials | Chen et al., 2016 |
| LG treatment as add-on to insulin improved HR and did not progress other cardiovascular risk factors after 6 months of treatment in patients with chronic T1D. | Dejgaard et al., 2017 |
| In insulin-treated patients with T2D, LG treatment may reduce visceral adiposity in parallel with the reduction of hepatic fat accumulation, albuminuria and micro-inflammation and progress quality of life associated with diabetes care. | Bouchi et al., 2017 |
| LG treatment might improve the therapeutic efficacy of mesenchymal stem cells in the treatment of T1D. | Li et al., 2016a |
| LG treatment decreased ostomy wet weight production in end-jejunostomy patients with SBS-IF and improved their intestinal wet weight and energy absorption. | Hvistendahl et al., 2016 |
| LG treatment delays onset of EAE in Lewis rats and is associated with improved protective capacity against oxidative stress. | Bouchi et al., 2017 |
| In failing post-ischemic T2D patients, LG therapy improves heart function and functional capacity. | Arturi et al., 2016 |
| LG improved heart rate and decreased heart rate variability despite weight loss and enhancement of metabolic parameters in overweight patients with CAD and newly diagnosed T2D. The rise in nocturnal heart rate in combination with a reduction in parasympathetic activity parameters proposes that LG may affect sympathovagal balance. | Kumarathurai et al., 2017b |
| Treatment of LG re-establishes angiogenesis in PA-impaired HUVECs via up-regulation of GTPCH1 and eNOS in a PI3K/Akt-Foxo1-dependent mechanism. | Ke et al., 2016 |
| Pre-treatment with LG reduced lipopolysaccharide-induced acute lung injury by preventing the NLRP3 inflammasome pathway. | Zhou et al., 2016 |
| LG has pleiotropic effects on renal risk factors. | Zobel et al., 2017 |
| Due to its safety and hypoglycemic efficacy, liraglutide is an excellent choice for DM treatment in combination with other drugs. Its effects on the reduction of weight and other cardiovascular risk factors, make it an optimal treatment, especially in overweight or obese patients. | Calvo Gomez et al., 2016 |
| Six months of treatment with liraglutide 1.2 mg/d significantly reduced LFC in patients with inadequately controlled type 2 diabetes and this effect was mainly driven by body weight reduction. | Petit et al., 2017 |
| In Indian T2D patients, treatment of LG resulted in a significant and continued decrease in HbA1c and body weight over a year and it was associated with baseline HbA1c and weight, respectively. | Kaur et al., 2016 |
| LG is safe and well tolerated for non-diabetic individuals with mood disorders as well as shows beneficial effects on objective measures of cognitive function. | Mansur et al., 2017 |
| LG might be moderate in the conformation of the gut microbiota, leads to a more lean-related profile that was consistent with its weight-losing effect. | Wang et al., 2016a |
| Through modification of AMPK/mTOR signaling, LG weakens osteoblastic differentiation of MC3T3 E1 cells. | Hu et al., 2016 |
| LG may have a beneficial role in pulmonary vascular remodeling, because of its defensive and therapeutic effects on monocrotaline-induced pulmonary arterial hypertension, through the eNOS/sGC/PKG and Rho kinase pathways. | Lee et al., 2016 |
| LG may hinder in the metabolism of energy because analysis of different times of administrations, concentrations, and level of brain development leads to different outcomes. | Pra et al., 2016 |
| The useful effects of LG on pancreatic islets seem to be linked to its anti-inflammatory and anti-oxidative properties. These conclusions showed that LG could be used to progress graft survival. | Langlois et al., 2016 |

Received: April 01, 2017, accepted: May 03, 2017, published: May 17, 2017

| Key Findings | References |
|---|---|
| LG in addition to insulin therapy declines HbA1c levels, total insulin dose, and body weight in a population that was usually demonstrative of subjects with T1D, together with increased rates of symptomatic hypoglycemia and hyperglycemia with ketosis, thereby limiting clinical use in this group. | Mathieu et al., 2016 |
| LG and Linagliptin through the initiation of the ERK/NF-κB/pathway, prevent glucose- and Ang II-induced collagen formation in cardiac fibroblasts. | Wang et al., 2016b |
| In T2D patients, LG therapy for 180 days is associated with a perfection in diastolic function. | Saponaro et al., 2016 |
| LG progresses endothelial function, weakens endothelial inflammatory signals, as well as converses leptin resistance. | Li et al., 2016b |
| In T2D patients, LG may be beneficial for treating insulin allergy and anti-insulin antibodies. | Htike et al., 2016 |
| Without modifying the level of plasma insulin, LG effectively enhanced glycemic instability due to insulin antibodies. | Kato et al., 2016 |
| Early use of LG may decrease metabolic insulin resistance and stop cardiovascular problems of diabetes. It exhibits their beneficial glycemic effect by improving microvascular insulin sensitivity and muscle capillary density during insulin resistance development. | Chai et al., 2016 |

## Figures and Tables

**Table 1:**
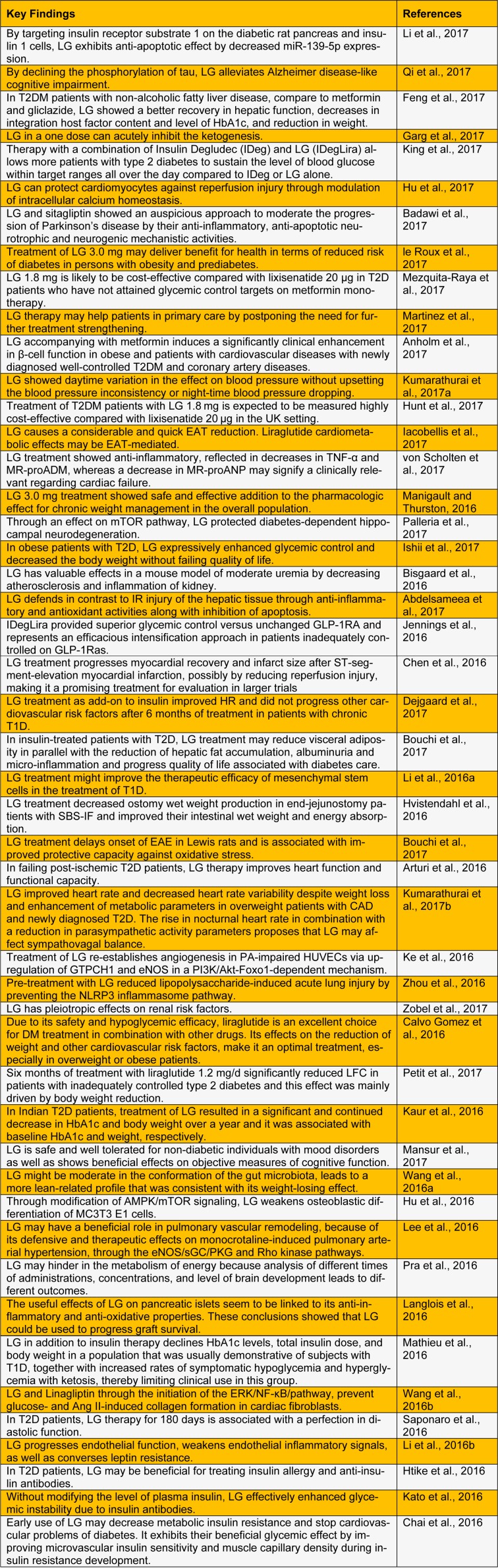
Recent update on biological activities and pharmacological actions of liraglutide
